# Antihypertensive strategies for the prevention of secondary stroke: a systematic review and meta-analysis

**DOI:** 10.1186/s40001-024-02226-3

**Published:** 2025-01-09

**Authors:** ChunQi Wang, Ling Feng, ShuangYan Tu, Dan Wei, Rui Wang, ZhiQiang Deng, YiPing Luo

**Affiliations:** 1 Department of Neurology, West China Hospital, Sichuan University, No. 37, Guoxue Lane, Wuhou District, Chengdu, 610041 Sichuan China; 2 West China School of Nursing, Sichuan University, Chengdu, 610041 Sichuan China; 3 Department of Liver Surgery, West China Hospital of Sichuan University, Chengdu, 610041 Sichuan China

**Keywords:** Stroke, Recurrent stroke, Hypertension, Blood pressure management, Antihypertensive therapy, Secondary prevention, Meta-analysis

## Abstract

**Background:**

Stroke is an important contributor to disability and death globally. Hypertension is a main risk factor for recurrent stroke in patients with ischemic and hemorrhagic stroke or transient ischemic attack. Higher systolic blood pressure, diastolic blood pressure, pulse pressure and mean arterial pressure at admission are independently associated with the risk of stroke recurrence. Therefore, lowering blood pressure is recommended by guidelines to prevent the recurrence of stroke.

**Methods:**

A systematic search of PubMed, Embase, Cochrane Central Register of Controlled Trials, and Web of Science databases was conducted through January 12, 2024. The search identified randomized controlled trials (RCTs) comparing antihypertensive drugs with control measures (placebo or no treatment) or standard blood pressure control (SBPC) with intensive blood pressure control (IBPC) for recurrent stroke prevention. Primary outcomes included overall and subtype stroke recurrence rates, fatal and non-fatal strokes, cardiovascular deaths, and myocardial infarctions (MIs). Secondary outcomes comprised non-fatal MIs and all-cause mortality. Risk ratios (RRs) and 95% confidence intervals (CIs) were calculated using random or fixed-effect models in Stata 15.0.

**Results:**

The analysis included 19 RCTs encompassing 72,048 patients. Twelve studies (n = 53,971) evaluated antihypertensive drugs against placebo or no treatment, while seven studies (n = 18,077) compared SBPC with IBPC. Antihypertensive therapy demonstrated significant risk reductions compared to placebo or no treatment for recurrent stroke (RR = 0.86, 95% CI: 0.75–0.97), cardiovascular deaths (RR = 0.92, 95% CI: 0.87–0.97), and MIs (RR = 0.87, 95% CI: 0.79–0.96). IBPC showed superior outcomes compared to SBPC, with significant reductions in recurrent stroke (RR = 0.87, 95% CI: 0.77–0.98), cardiovascular deaths (RR = 0.75, 95% CI: 0.61–0.91), and all-cause mortality (RR = 0.85, 95% CI: 0.73–0.95).

**Conclusion:**

In stroke patients, antihypertensive therapy demonstrates significant protective effects against stroke recurrence, cardiovascular deaths, and MIs compared to placebo or no treatment. Additionally, IBPC provides enhanced protection against stroke recurrence, cardiovascular deaths, and all-cause mortality compared to SBPC.

**Supplementary Information:**

The online version contains supplementary material available at 10.1186/s40001-024-02226-3.

## Introduction

Stroke is an important contributor to disability and death globally [1]. More than half of stroke-related deaths are related to high blood pressure, and lowering blood pressure can reduce the risk of stroke or recurrence in old patients [2]. Global statistics from 2019 position stroke as the second leading cause of mortality and disability worldwide [3–5]. Hypertension represents a significant risk factor contributing to stroke recurrence, dementia, and other blood disorders [6]. Clinical management of blood pressure in stroke patients primarily focuses on two strategies: antihypertensive medication and intensive blood pressure control (IBPC). Standard pharmacological interventions include several drug classes: angiotensin II receptor antagonists (ARAs, including candesartan and telmisartan), angiotensin-converting enzyme inhibitors (ACEIs, such as perindopril and ramipril), thiazide diuretics (indapamide), and β1-adrenergic receptor blockers (atenolol). IBPC protocols typically aim to maintain systolic blood pressure (SBP) below 130/85 mmHg. The SPS3 trial conducted in the United States compared different SBP targets. Results demonstrated that maintaining SBP below 130 mmHg significantly reduced cardiovascular events in patients with cerebrovascular disease [7]. Mant et al.’s research subsequently confirmed these findings [8]. The SPRINT trial further reported decreased major cardiovascular events and mortality rates when targeting SBP below 120 mmHg. However, Kitagawa et al.’s study revealed that while IBPC reduced both annual stroke recurrence and hemorrhagic stroke risk, it showed no significant effect on ischemic stroke risk [9]. Given these conflicting findings, the efficacy of blood pressure control strategies in improving stroke prognosis remains a subject of debate.

A 2017 meta-analysis by Zonneveld et al. examined 11 trials: eight investigating antihypertensive drugs and three comparing blood pressure targets [6]. Their findings indicated that antihypertensive medications reduced stroke recurrence risk in patients with stroke or transient ischemic attack (TIA), although IBPC showed only modest benefits. The latest study included in the analysis by Zonneveld et al. [6] was published in 2017. Their results indicate that antihypertensive drugs are not effective in reducing cardiovascular events, different stroke types, myocardial infarction, death from any cause, and blood pressure compared with placebo or no treatment with antihypertensive drugs. Therefore, this study aimed to more comprehensively analyze the above outcomes and update previous meta-analyses, in order to provide a reference for future research.

## Methods

### Registration statement

The study protocol followed the Preferred Reporting Items for Systematic Reviews and Meta-Analyses (PRISMA) guidelines. The review was registered on PROSPERO with registration number CRD42023404180.

### Study selection

#### Inclusion criteria

The meta-analysis inclusion criteria encompassed studies with participants having a history of stroke or TIA. Two intervention types were considered: antihypertensive drugs vs. placebo/no treatment, or IBPC (target <130/85 mmHg) vs. standard blood pressure control (SBPC, target <140/90 mmHg). Primary outcomes comprised recurrent strokes (ischemic and hemorrhagic), fatal stroke, non-fatal stroke, cardiovascular death, and myocardial infarction (MI). Secondary outcomes included non-fatal MI and all-cause mortality. Only RCTs with follow-up data were included.

#### Exclusion criteria

The analysis excluded reviews, meta-analyses, guidelines, abstracts, and conference papers. Additional exclusions included case reports, letters, cohort studies, other observational studies, and studies lacking full texts or essential data.

### Search methods and resources

A systematic search was conducted across PubMed, Cochrane Central Register of Controlled Trials, Embase, and Web of Science databases for relevant English publications. The initial search concluded on February 20, 2023, with an update on January 12, 2024. The search strategy incorporated both MeSH terms and free-text terms. Reference lists of reviews and meta-analyses underwent additional screening to ensure comprehensive coverage.

### Literature screening results

**Fig. 1:**
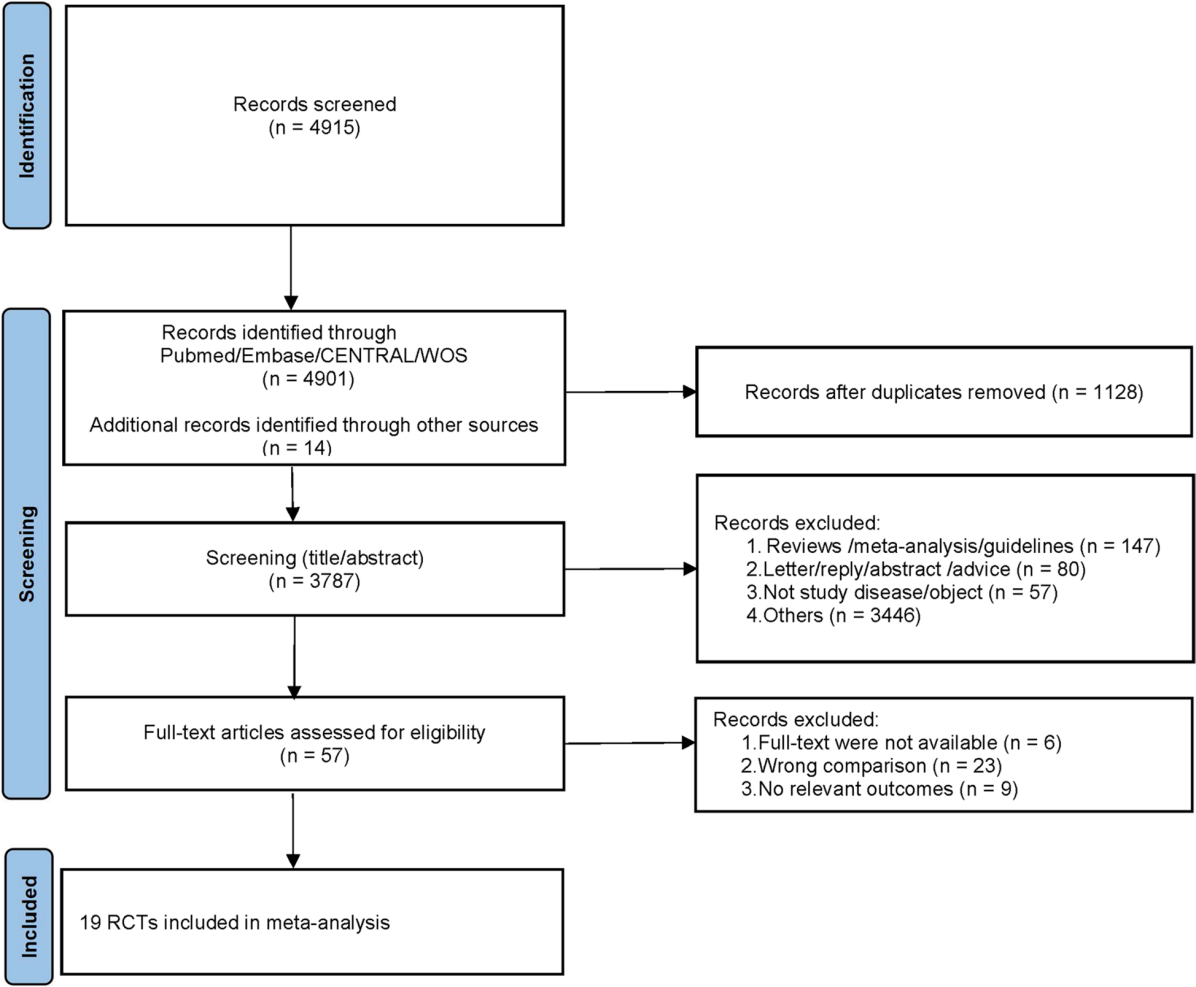
Study selection process and results

The initial database search identified 4915 papers. After removing duplicates, 3787 titles and abstracts underwent screening, resulting in 3731 exclusions based on inclusion criteria. Full-text review of 56 articles led to the final selection of 19 studies for meta-analysis (as illustrated in Fig. 1).

### Data extraction

A standardized form facilitated the extraction of comprehensive study data. The extracted information included publication details (authors, year, country), methodological characteristics (study design, patient source, inclusion criteria), participant demographics (sample size, gender distribution, age), intervention specifics (antihypertensive and control strategies), clinical parameters (stroke type, disease background), and outcome data (primary measures, follow-up duration). Additional information was obtained through author correspondence when necessary. The reasons for trial exclusion were systematically documented.

### Quality evaluation

Two independent reviewers (WCQ and DZQ) assessed the risk of bias using the Cochrane ROB2 Tool. The assessment covered five domains: randomization process, intervention deviations, missing outcome data, outcome measurement, and selective reporting. Each study received a risk classification of low, unclear, or high. A senior reviewer (FL) resolved any disagreements through discussion and consultation.

### Outcome measures

The study evaluated both primary and secondary outcomes. Primary outcomes encompassed overall and subtype stroke recurrence rates, fatal and non-fatal strokes, cardiovascular deaths, and MIs. Secondary outcomes comprised non-fatal MIs and all-cause mortality.

### Data synthesis

STATA 15.0 facilitated the statistical analysis. Risk ratio (RR) served as the effect measure for dichotomous variables. The Q test assessed inter-study heterogeneity (α = 0.10 for significance), with *P* < 0.10 indicating significant heterogeneity. The *I*^2^ statistic quantified heterogeneity levels: 25, 50, and 75% representing low, moderate, and high heterogeneity, respectively. Studies with high heterogeneity (*I*^2^ > 50%) warranted a random-effects model; otherwise, a fixed-effects model was applied. Subgroup and regression analyses explored heterogeneity sources. Sensitivity analyses confirmed result stability and reliability. Publication bias assessment employed funnel plots, Begg’s test, and Egger’s test, with the trim-and-fill method evaluating bias impact on results.

## Results

### Literature screening results

Detailed search strategies are documented in Table S1. The systematic search and screening process is illustrated in Fig. 1.

### Characteristics of included studies

The meta-analysis incorporated nineteen studies [5–7, 9–22, 24] with 72,408 participants from seven countries (China, Norway, USA, UK, Australia, Japan, Canada). The study population comprised 11,242 women (17%) and 60,804 men (83%), aged between 59 and 73 years. Follow-up averaged 3.2 years across all studies. Each study demonstrated comparable baseline characteristics between groups and employed intention-to-treat analysis. Among the included studies, twelve trials (n = 53,971) evaluated antihypertensive drugs against placebo or no treatment, while seven trials (n = 18,077) compared IBPC with SBPC (Table 1).

**Table 1:** Basic characteristics of the included studies

Study (year)	Study type	Country*	Sample size (T/C)	Mean age (T/C, year)	Gender (male/female)		Intervention	
					T	C	T	C
Sandset et al. (2011) [11]	RCT	Norway	1017/1012	70.8 ± 11.2/71.0 ± 11.0	612/405	564/448	Candesartan	Placebo
PROGRESS (2001) [12]	RCT	Australia	3051/3054	64.0 ± 10.0/64.0 ± 10.0	2128/923	2125/929	Perindopril + Indapamide	Placebo
Dutch TIA (1993) [13]	RCT	The Netherlands	732/741	54/50	66/666	62/679	Atenolol	Placebo
Bath et al. (2017) [24]	RCT	Britain	41/42	73.0 ± 6.5/75.1 ± 6.9	33/8	31/11	Intensive antihypertensive therapy	Standard antihypertensive therapy
Kitagawa et al. (2019) [9]	RCT	Japan	633/630	67.2c8.8/67.3 ± 8.8	449/184	428/202	Intensive antihypertensive therapy	Standard antihypertensive therapy
Mant et al. (2016) [8]	RCT	Britain	266/263	71.9 ± 9.1/71.7 ± 9.7	157/109	156/107	Intensive antihypertensive therapy	Standard antihypertensive therapy
SPS3 (2013) [7]	RCT	American	1501/1519	63 ± 10.7/63 ± 10.8	990/511	912/607	Intensive antihypertensive therapy	Standard antihypertensive therapy
Liu et al. (2009) [15]	RCT	China	2840/2825	60.1 ± 8.3/60.4 ± 8.5	2037/803	2040/785	Indapamide	Placebo
SPRINT (2015) [16]	RCT		4678/4683	67.9 ± 9.4/67.9 ± 9.5	2994/1684	2999/1684	Intensive antihypertensive therapy	Standard antihypertensive therapy
Yusuf et al. (2000) [17]	RCT	Canada	4645/4652	66 ± 7	3366/1279	3451/1201	Ramipril	Placebo
PATS (1995) [18]	RCT	China	2824/2824	60 ± 8	72/2752	72/2752	Indapamide	Placebo
Hypertension-Stroke (1974) [19]	RCT	American	232/219	59	139/94	126/93	Deserpidine + Methyclothiazide	Placebo
Yusuf et al. (2008) [20]	RCT	Canada	10,146/10186	66.1 ± 8.6/66.2 ± 8.6	6527/3619	6567/3619	Telmisartan	Placebo
Hasegawa et al. (2004) [21]	RCT	Japan	328/339	60 ± 9.0	244/84	254/85	Perindopril	Placebo
Odden et al. (2016) [22]	RCT	American	747/2000	63.8 (10.5)/63.2 (10.7)	462/285	1278/722	Intensive antihypertensive therapy	Standard antihypertensive therapy
Olofsson et al. (1995) [25]	RCT	Sweden	372/348	70.1 ± 8.6/70.7 ± 9.1	212/160	220/128	Atenolol	Placebo
Carter (1970) [23]	RCT		50/49	Below 80	27/23	29/20	Combination of methyldopa, betanidine, or amodiaquine to limit salt intake, weight loss, and thiazide diuretics	Placebo
Bath et al. (2009) [14]	RCT	Britain	647/713	66.8 (8.8)/67.1 (9.2)	420/227	464/249	Telmisartan	Placebo
Kitagawa et al. (2022) [26]	RCT	Japan	532/542	67.8	155/377	174/368	Intensive antihypertensive therapy	Standard antihypertensive therapy

Study (year)	Follow-up time (year)	Baseline SBP (T/C, mmHg)	Baseline DBP (T/C, mmHg)	Target SBP (T/C, mmHg)	Target DBP (T/C, mmHg)	End SBP (T/C, mmHg)	Outcome indicators
Sandset et al. (2011) [11]	0.5	171.2/171.6	90.3/90.6	NR	NR	141/141	1, 4, 5, 6
PROGRESS (2001) [12]	4	147/147	86/86	NR	NR	135/144	1, 3, 4, 5, 6
Dutch TIA (1993) [13]	2.6	158/157	91/91	NR	NR	149/159	1, 2, 3, 4, 5, 6
Bath et al. (2017) [24]	2	145.9/148.3	82.5/81.7	<125/<140	NR	NR	1, 5
Kitagawa et al. (2019) [9]	6.2	145.1/145.7	83.6/83.7	126.7/133.2	77.4/77.7	123.7/132.0	1, 2, 3, 4, 5, 6
Mant et al. (2016) [8]	1	143.5/142.2	78.8/80.7	<125/<140	NR	127.4/129.4	1, 2, 3, 5, 6
SPS3 (2013) [7]	3.7	144/142	7 9/78	<130/<130 ~ 149	NR	127/138	1, 5, 6
Liu et al. (2009) [15]	1	154.0/153.6	93.0/92.6	147.2/146.8	89.7/89.3	141.4/146.9	1, 2, 3, 5, 6
SPRINT (2015) [16]	3.26	139.7/139.7	78.2/78.0	<120/<140	NR	121.5/134.6	1, 5, 6
Yusuf et al. (2000) [17]	5	139/139	79/79	NR	NR	136/139	1, 5, 6
PATS (1995) [18]	2	154.0/153.6	93.0/92.6	NR	NR	141.4/1 46.9	1, 5, 6
Hypertension-Stroke (1974) [19]	5	167/167	100/100	NR	NR	142/167	1, 2, 3, 5, 6
Yusuf et al. (2008) [20]	2.5	144.1/144.2	84/84	NR	NR	137/141	1, 4, 5
Hasegawa et al. (2004) [21]	3.9	143/143	84/83	137.8/137.8	81.4/80.4	NR	1
Odden et al. (2016) [22]	3.7	135/146	75/80	<130/<130 ~ 149	NR	126/137	1, 4, 5, 6
Olofsson et al. (1995) [25]	2.3	161/161	88/89	NR	NR	157/161	1, 3, 5, 6
Carter (1970) [23]	4	>160/>160	>110/>110	<160/<160	90–100/90–100	NR	1, 2, 3, 4, 6
Bath et al. (2009) [14]	2.5	146/147	84/84	NR	NR	135/141	1, 3, 4, 5, 6
Kitagawa et al. (2022) [26]	3.9	139.8/141.5	81.1/81.6	<120/<140	<80/<90	126.7/133.4	1

*Country of the first author’s institution

*RCT* randomized controlled clinical trial, *T* test group, *C* control group, *SBP* systolic blood pressure, *DBP* diastolic blood pressure. (1) stroke recurrence; (2) fatal and disabling stroke; (3) non-fatal and disabling stroke; (4) vascular death; (5) non-fatal myocardial infarction; (6) all-cause mortality

### Risk of bias assessment

**Fig. 2:**
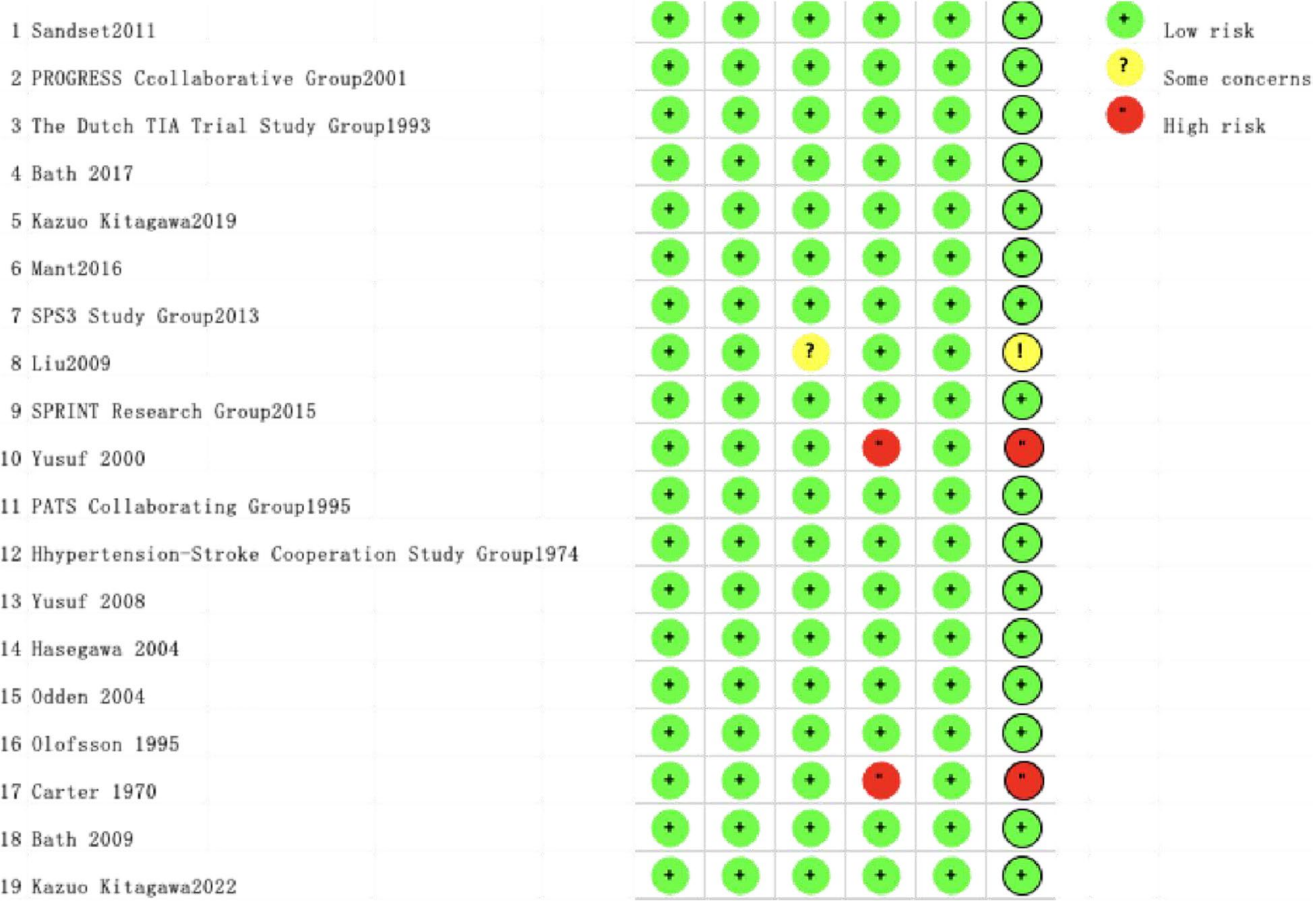
Risk of bias assessment (1)

**Fig. 3:**
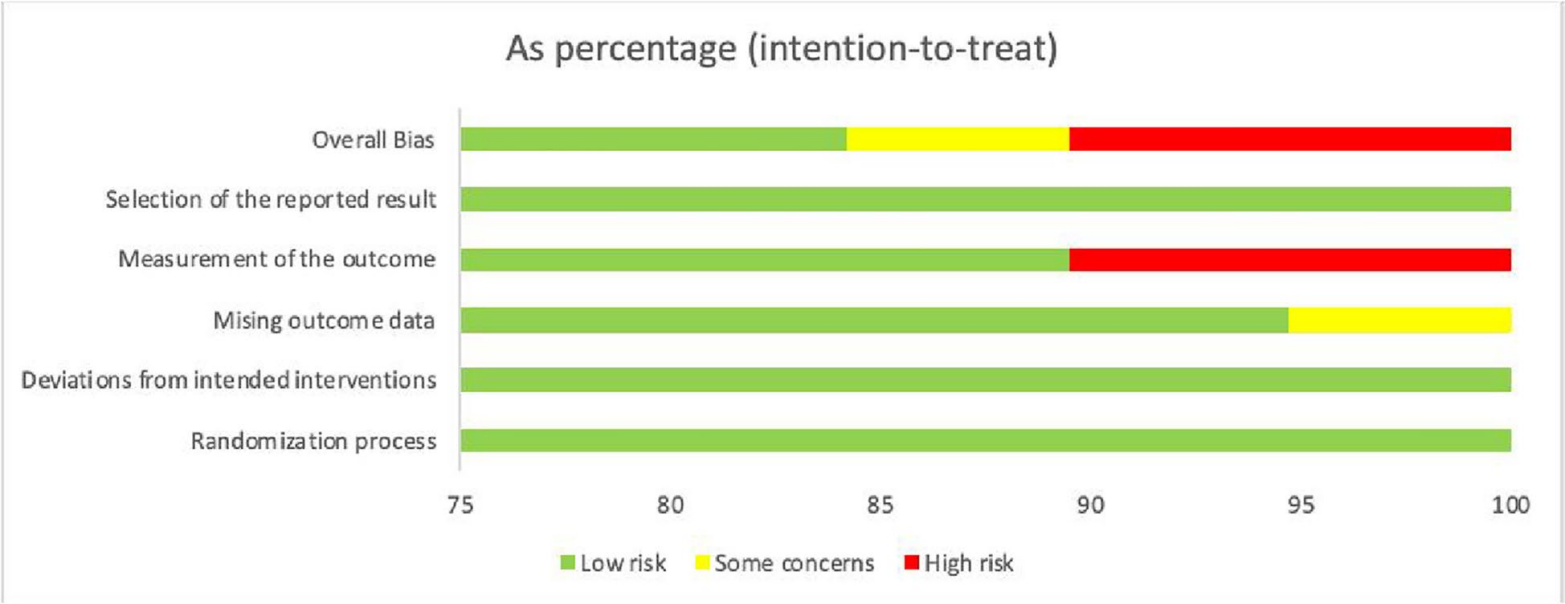
Risk of bias assessment (2)

The quality assessment revealed low risk of bias in sixteen studies, medium risk in one study due to missing outcome data, and high risk in two studies stemming from outcome measurement and result reporting biases. Figures 2 and 3 present the comprehensive risk assessment outcomes.

### Antihypertensive drugs vs. placebo or no treatment

#### Recurrent stroke

**Fig. 4:**
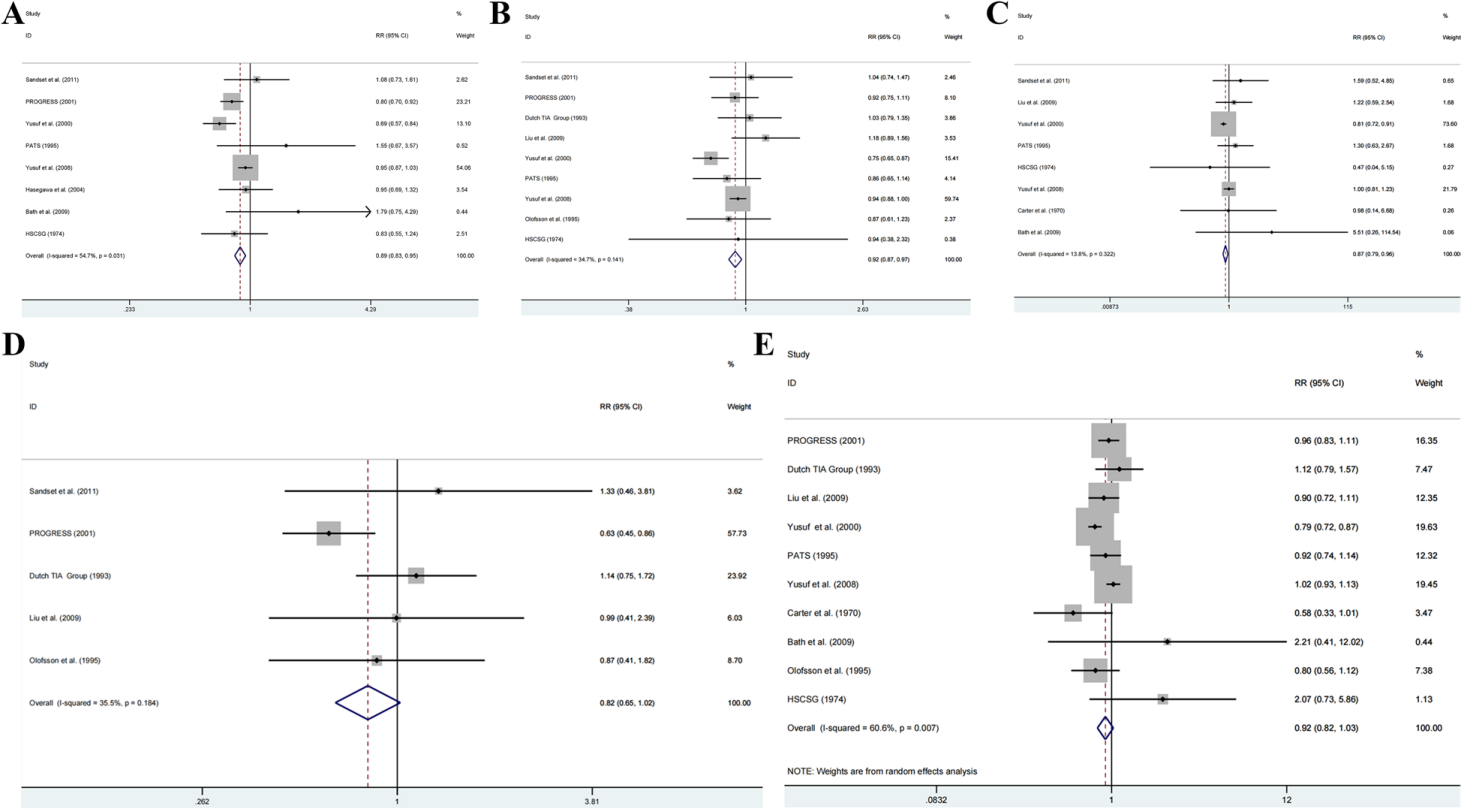
Forest plot for **A** recurrent stroke, **B** cardiovascular death, **C** MI, **D** non-fatal MI, **E** all-cause mortality between antihypertensive drugs and placebo or no treatment

A meta-analysis of eight studies (46,255 patients) examined the impact of antihypertensive drugs on stroke recurrence [11, 12, 14, 17–21]. Moderate heterogeneity existed among studies (*I*^2^ = 54.7%, *P* = 0.031), necessitating a random effects model. Analysis revealed that antihypertensive drugs significantly reduced recurrent stroke risk compared to placebo or no treatment (RR = 0.89, 95% CI: 0.83–0.95) (Fig. 4A).

#### Cardiovascular death

Nine studies (51,738 patients) investigated antihypertensive drug effects on cardiovascular mortality [11–13, 15, 17–20, 25]. With acceptable heterogeneity (*I*^2^ = 34.7%, *P* = 0.141), a fixed effect model demonstrated significant cardiovascular death risk reduction with antihypertensive treatment (RR = 0.92, 95% CI: 0.87–0.97) (Fig. 4B).

#### MI

Analysis of eight studies (44,899 patients) [11, 14, 15, 17–20, 23] using a fixed effect model showed significantly decreased MI risk in the antihypertensive group (*I*^2^ = 13.8%, *P* = 0.322; RR = 0.87, 95% CI: 0.79–0.96) (Fig. 4C).

#### Non-fatal MI

Five studies (15,992 patients) [11–13, 15, 25] examined non-fatal MI outcomes. Using a fixed-effects model (*I*^2^ = 35.5%, *P* = 0.184), analysis showed no significant risk reduction with antihypertensive treatment (RR = 0.82, 95% CI: 0.65–1.02) (Fig. 4D).

#### All-cause mortality

Ten studies (51,161 patients) [12–15, 17–20, 23, 25] evaluated all-cause mortality. A random effects model (*I*^2^ = 60.6%, *P* = 0.007) revealed a non-significant trend toward lower mortality risk with antihypertensive treatment (RR = 0.92, 95% CI: 0.82–1.03) (Fig. 4E).

#### Subgroup analysis and regression analysis of antihypertensive drugs vs. placebo or no treatment

**Fig. 5:**
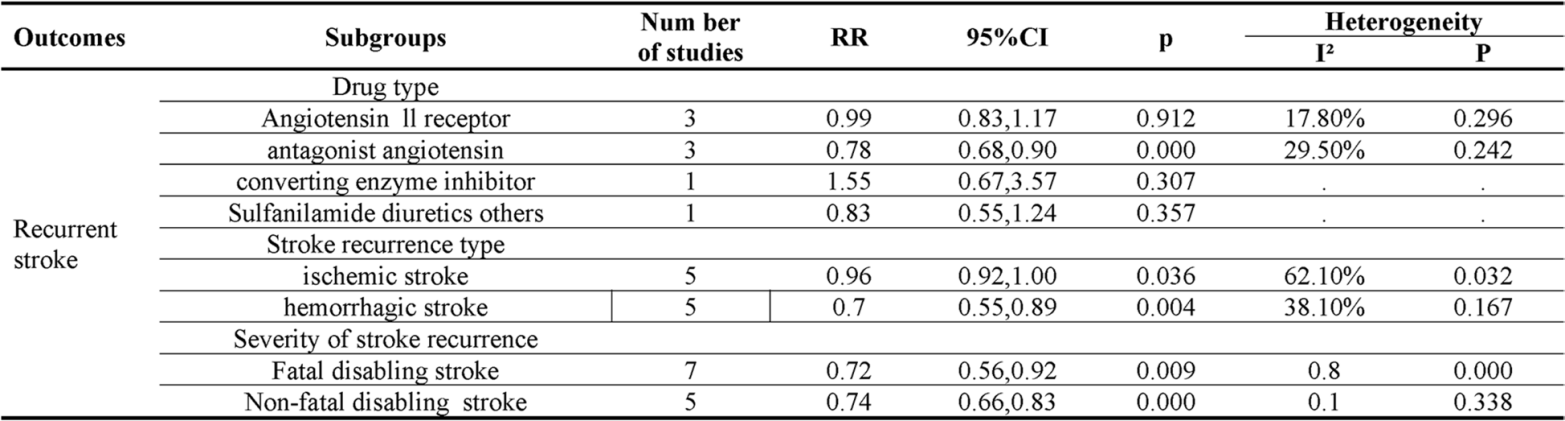
Subgroup and regression analysis of antihypertensive therapy vs. control

Subgroup analyses examined heterogeneity sources based on drug types, recurrent stroke characteristics, and stroke severity (Fig. 5). Analysis by drug type revealed that ACEIs significantly reduced stroke recurrence risk (RR = 0.78, 95% CI: 0.68–0.90). However, sulfanilamide diuretics and ARAs showed no significant preventive effect (RR = 0.99, 95% CI: 0.83–1.18; RR = 1.55, 95% CI: 0.67–3.57, respectively). Similarly, combination antihypertensive therapy demonstrated no significant reduction in stroke recurrence risk (RR = 0.83, 95% CI: 0.55–1.24). Analysis by stroke type demonstrated that while antihypertensive drugs did not significantly prevent ischemic stroke (RR = 0.96, 95% CI: 0.92–1.00), they markedly reduced hemorrhagic stroke risk (RR = 0.70, 95% CI: 0.55–0.89). Regarding stroke severity, antihypertensive treatment effectively reduced both fatal disabling stroke (RR = 0.72, 95% CI: 0.56–0.92) and non-fatal disabling stroke risks (RR = 0.74, 95% CI: 0.66–0.83).

Further regression analysis was performed based on the outcome indicators of antihypertensive drugs and placebo or no use of antihypertensive drugs. The results showed that drug type may be the source of heterogeneity (*P* < 0.01). Detailed resulst are provided in Table S2.

### Meta-analysis: intensive vs. standard blood pressure lowering

#### Recurrent stroke

**Fig. 6:**
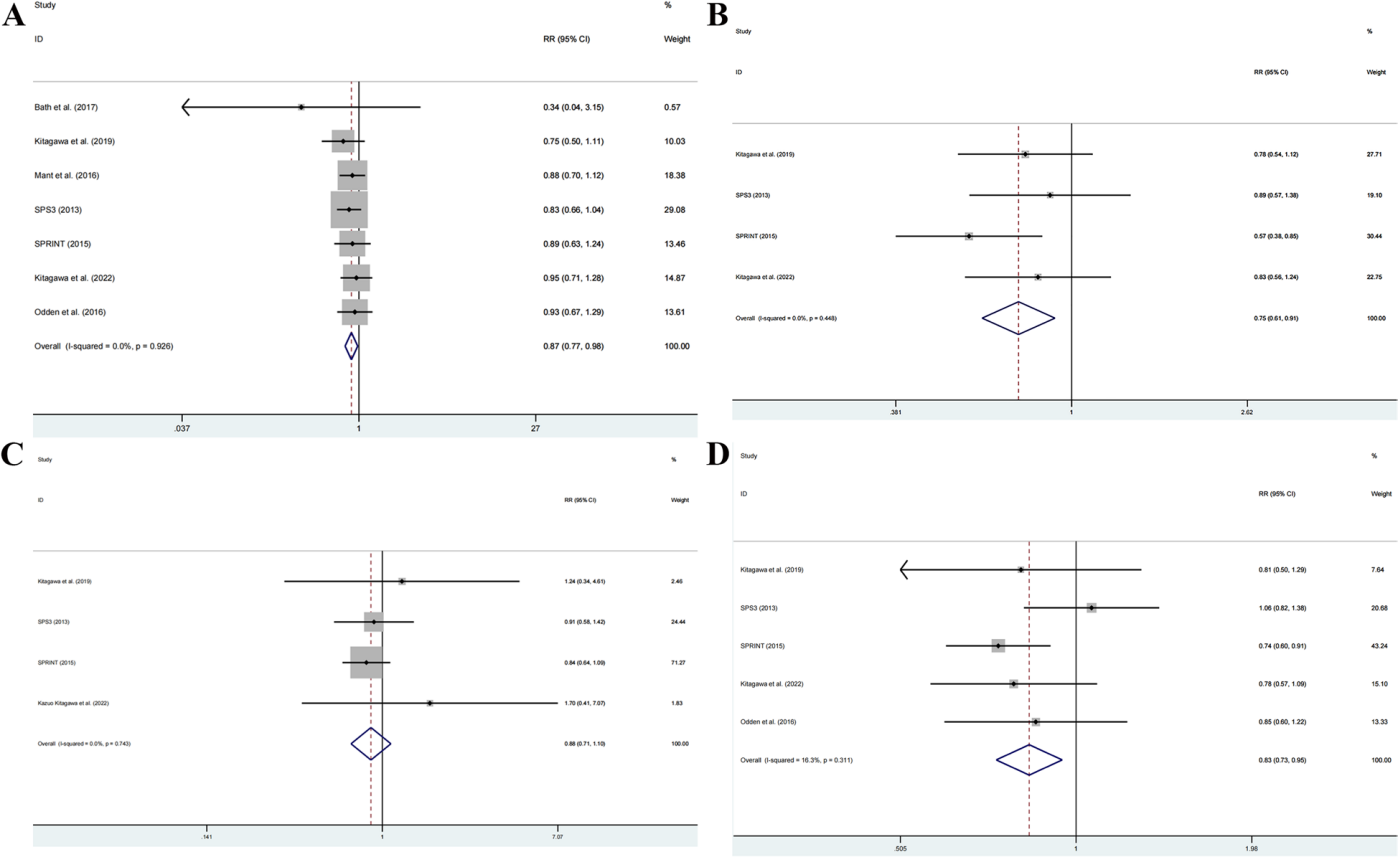
Forest plot of **A** recurrent stroke, **B** cardiovascular death, **C** MI, **D** all-cause mortality between IBPC and SBPC

Analysis of seven studies (18,077 patients) examined the effects of intensive blood pressure lowering on stroke recurrence [7–9, 16, 18, 22, 26]. No significant heterogeneity existed (*I*^2^ = 0.0%, *P* = 0.926). Using a fixed-effect model, IBPC significantly reduced recurrent stroke risk compared to SBPC (RR = 0.87, 95% CI: 0.77–0.98) (Fig. 6A).

#### Cardiovascular death

Analysis of four studies (7747 patients) evaluated cardiovascular mortality outcomes [7, 9, 16, 26]. With no heterogeneity (*I*^2^ = 0.0%, *P* = 0.448), IBPC significantly reduced cardiovascular death risk compared to SBPC (RR = 0.75, 95% CI: 0.61–0.91) (Fig. 6B).

#### MI

Analysis of four studies (14,718 patients) examined the effects of intensive blood pressure lowering on MI [7, 9, 16, 26]. No significant heterogeneity existed (*I*^2^ = 0.0%, *P* = 0.743). Fixed-effect model analysis revealed no significant difference between IBPC and SBPC in MI risk (RR = 0.88, 95% CI: 0.71–1.10) (Fig. 6C).

#### All-cause mortality

Analysis of five studies (17,465 patients) evaluated the effects of intensive blood pressure lowering on all-cause mortality [7, 9, 16, 22, 26]. Using a fixed-effect model (*I*^2^ = 16.3%, *P* = 0.311), results showed significantly reduced all-cause mortality risk with IBPC compared to SBPC (RR = 0.83, 95% CI: 0.73–0.95) (Fig. 6D).

#### Subgroup analysis

**Fig. 7:**
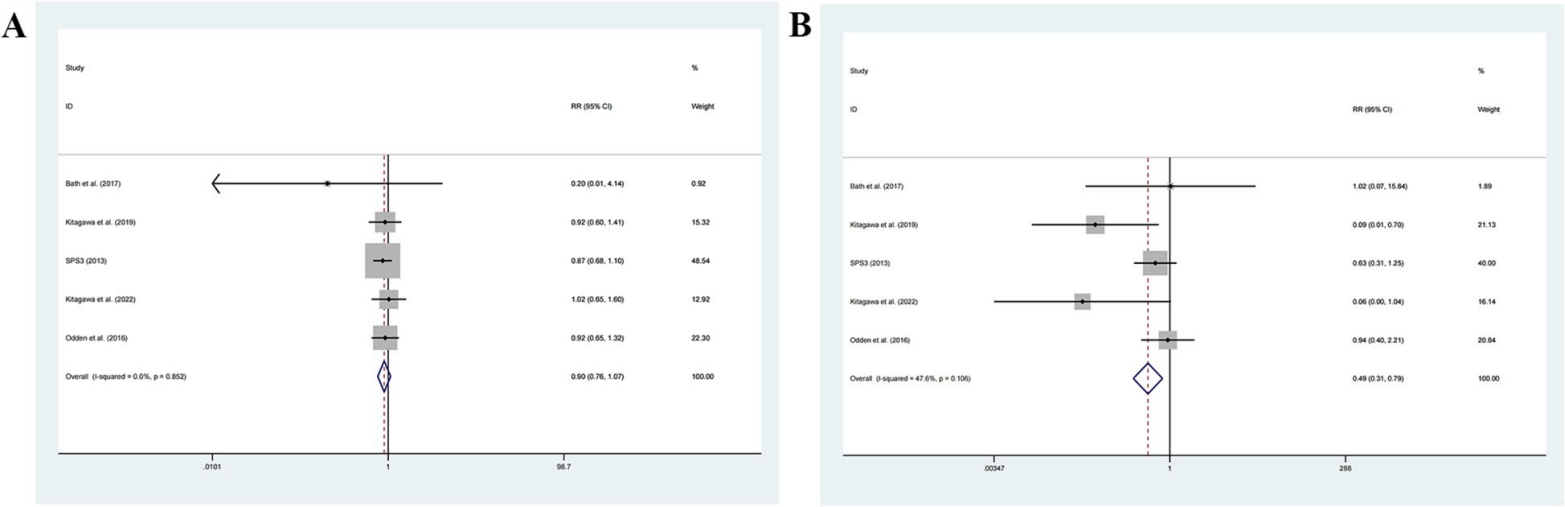
Forest plot of **A** recurrent ischemic stroke, **B** recurrent hemorrhagic stroke between IBPC and SBPC

Subgroup analyses explored heterogeneity sources based on IBPC targets and recurrent stroke types (Fig. 7). Different IBPC targets (<120 mmHg, ≤126 mmHg, <130 mmHg SBP) showed no significant reduction in overall recurrent stroke risk compared to SBPC (RR = 0.92, 95% CI: 0.74–1.15; RR = 0.83, 95% CI: 0.67–1.02; RR = 0.86, 95% CI: 0.72–1.04, respectively). Further subgroup analysis based on the type of recurrent strokes revealed no significant reduction in the risk of ischemic strokes (RR = 0.90, 95% CI: 0.76–1.07) (Fig. 7A) but a significant reduction in the risk of hemorrhagic apoplexy (RR = 0.49, 95% CI: 0.31–0.79) (Fig. 7B).

### Sensitivity analysis

**Fig. 8:**
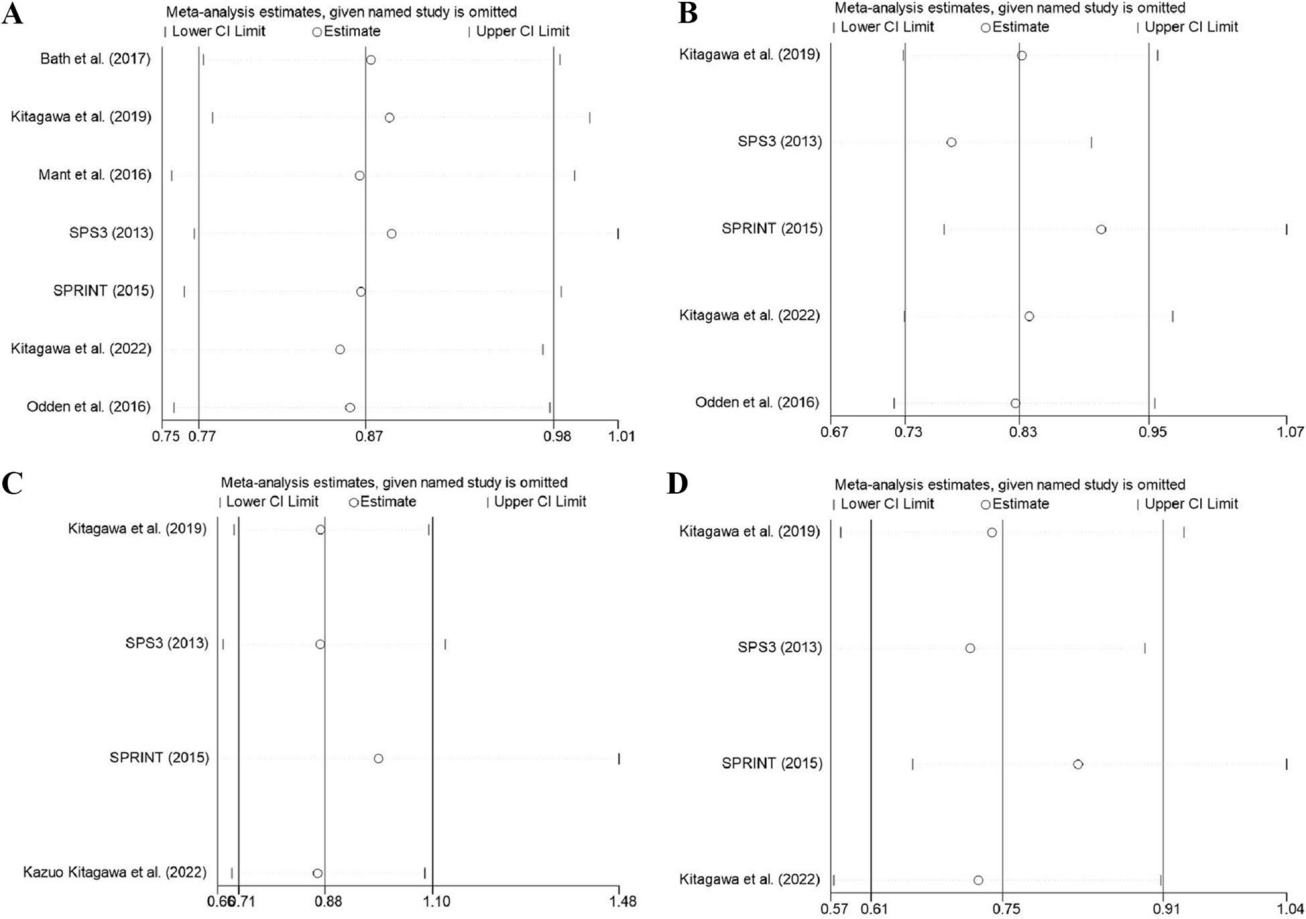
Sensitivity analysis of IBPC and SBPC. **A** recurrent stroke, **B** all-cause mortality, **C** MI, **D** cardiovascular death

**Fig. 9:**
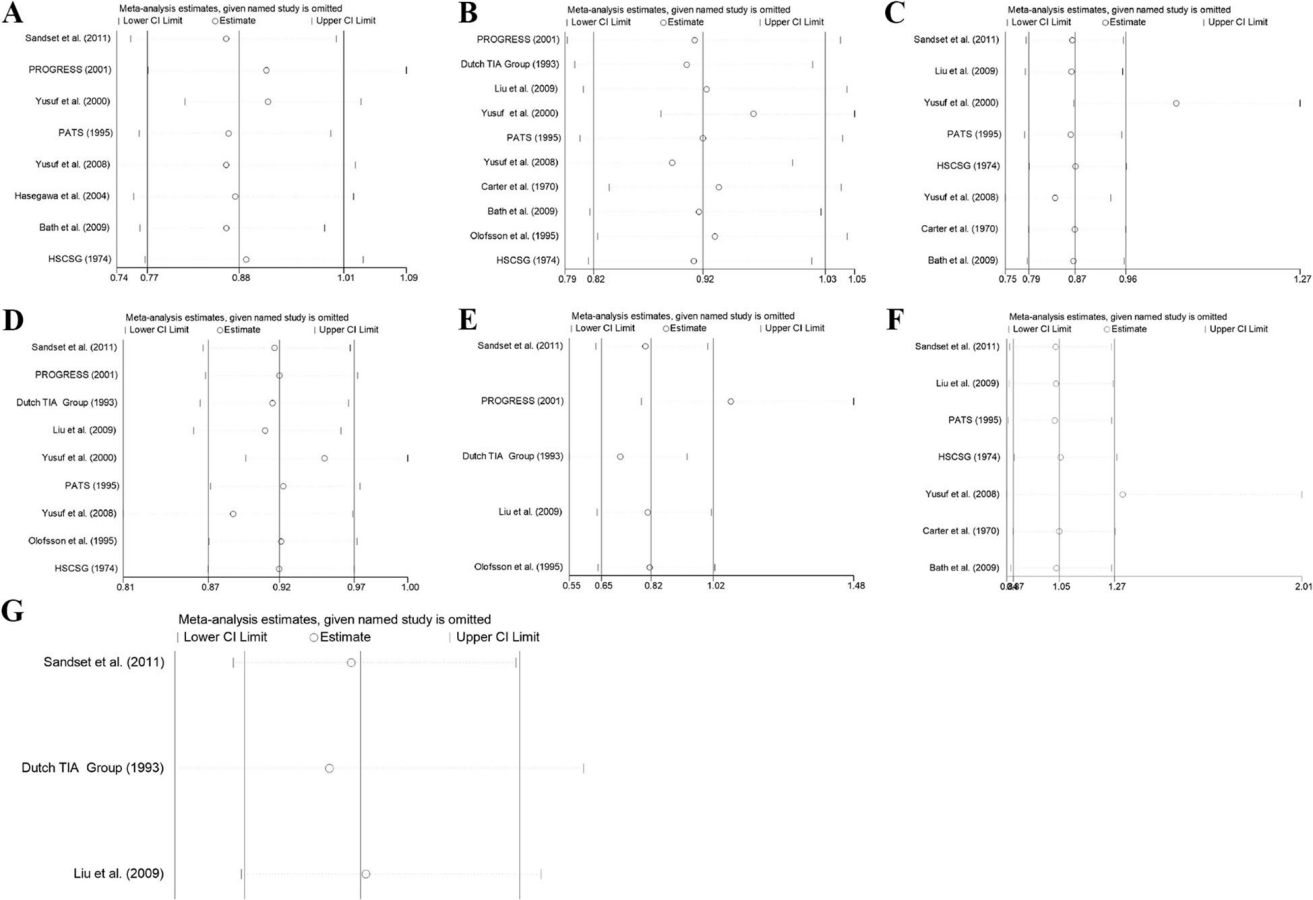
Sensitivity analysis of antihypertensive therapy vs. control. **A** recurrent stroke, **B** all-cause mortality, **C** MI, **D** cardiovascular death, **E** non-fatal MI, **F** MI (exclude Yusuf et al. [17]), **G** non-fatal MI (exclude PROGRESS [12])

The results of recurrent stroke, all-cause death, myocardial infarction, and cardiovascular death were generally stable in the intensive and standard blood pressure lowering groups (Fig. 8). The results of recurrent stroke, all-cause death, and cardiovascular death were generally stable in the antihypertensive drug and placebo or non-antihypertensive drug groups, while the results of non-fatal myocardial infarction were unstable. After removing the study of PROGRESS [12], the heterogeneity statistic of *I*^2^ declined from 35.5 to 0.00%, indicating that the study may be the source of heterogeneity, as shown in Fig. 9F. The results of myocardial infarction in the antihypertensive drug and placebo or non-antihypertensive drug groups were unstable. After removing the study of Yusuf et al. [17], the heterogeneity statistic of *I*^2^ declined from 13.8 to 0.00%, and the meta-analysis results changed from (*I*^2^ = 13.8%, *P* = 0.322; RR = 0.87, 95% CI: 0.79–0.96) to (*I*^2^ = 0.00%, *P* = 0.827; RR = 1.05, 95% CI: 0.87–1.27), with no statistic difference. Therefore, it may be a source of heterogeneity and also affect the stability of the results, as shown in Fig. 9G. The results of this outcome should be interpreted with caution. The results are shown in Figs. 8A–D and 9A–G.

### Publication bias

Publication bias assessment employed funnel plots combined with Egger’s and Begg’s tests for primary outcome measures. We analyzed publication bias for the outcome indicators reported in more than 10 studies. The results showed that the *P* value for all-cause death was 0.589, and there was no obvious publication bias. Relevant results of publication bias are shown in Table S3.

## Discussion

This meta-analysis evaluated 19 studies encompassing 72,048 individuals with stroke or TIA history. The findings demonstrate significant risk reductions with antihypertensive therapy compared to placebo or no treatment for stroke recurrence, cardiovascular death, and MI. Additionally, IBPC showed superior outcomes vs. SBPC in preventing stroke recurrence, cardiovascular death, and all-cause mortality.

### Antihypertensive drugs vs. placebo or no treatment

Antihypertensive therapy demonstrated significant efficacy in reducing stroke recurrence risk (RR = 0.86, 95% CI: 0.75–0.97). Different antihypertensive drug classes showed varying effectiveness. ACEIs, either alone or in combination, markedly reduced stroke recurrence risk. However, sulfanilamide diuretics or ARAs alone showed no significant preventive effect. These findings align with previous research [10]. Findings variations across studies likely stem from differences in baseline patient characteristics, antihypertensive drug selection, stroke recurrence definitions, and follow-up duration. The Dutch TIA trial [13] found no significant reduction in MI rates with post-stroke atenolol treatment, possibly due to limited sample size (372 experimental vs. 348 placebo) and modest drug dosage (50 mg atenolol). The PROGRESS study findings differed from Yusuf's 2008 research [12, 20] regarding telmisartan's effectiveness. PROGRESS participants showed higher baseline blood pressure (147/86 vs. 144/84 mmHg in Yusuf's study). Additionally, 58% of PROGRESS participants received combined perindopril-indapamide therapy, achieving greater blood pressure reduction compared to ACEI monotherapy (12.3/5.0 vs. 4.9/2.8 mmHg). Study duration also differed significantly (4 years for Yusuf vs. 2.5 years for PROGRESS). The risk of cardiovascular death significantly declines in the antihypertensive drug group, consistent with findings reported in a previous meta-analysis [10, 27]. Our results show that the risk of myocardial infarction significantly decreases in the antihypertensive drug group (RR = 0.87, 95% CI: 0.79–0.96), which is consistent with the results of previous studies [28, 29].

Antihypertensive therapy showed no significant reduction in non-fatal MI risk among stroke patients (RR = 0.82, 95% CI: 0.65–1.02), aligning with previous findings [13, 15]. Similarly, no significant impact on all-cause mortality was observed, potentially due to limited sample size.

A key study by Olofsson [13] demonstrated notable limitations in atenolol's effectiveness. Initially targeting 1900 patients with a two-year follow-up, the study ultimately included only 720 patients, with variable recruitment rates across centers. The results showed no significant prevention of vascular-related death, non-fatal stroke, or non-fatal MI in TIA or non-disabling ischemic stroke patients. Several factors may explain these inconsistent outcomes. The limited participant numbers potentially obscured atenolol's benefits. Additionally, the study population showed lower vascular event risk compared to excluded individuals with cardiac embolism sources, existing β-blocker use, or β-blocker contraindications (diabetes, congestive heart disease). Furthermore, hypertension history varied significantly between studies, with only 25% in the atenolol study vs. 50% in the aspirin study. The efficacy of β-blockers appears influenced by type and dosage considerations. While 50 mg atenolol demonstrated significant antihypertensive effects, higher doses were avoided to minimize preventive medication side effects. The underlying pharmacological mechanisms of β-blockers' post-MI benefits remain unclear. Despite significant heterogeneity in recurrent stroke and cardiovascular death risks, sensitivity analysis excluding the most influential study showed stable and reliable results. While these findings support blood pressure management for secondary stroke prevention, the specific advantages of pharmacological intervention beyond blood pressure reduction require further investigation through additional trials.

### IBPC versus SBPC

The meta-analysis results demonstrate that IBPC effectively reduces multiple risks: recurrent stroke, cardiovascular death, all-cause mortality, and hemorrhagic stroke. Varying conclusions across studies [7, 8] likely stem from differences in follow-up duration and blood pressure targets. While IBPC shows no statistically significant advantage over SBPC for several outcomes, its primary benefit appears to be reduced recurrent stroke risk, with less impact on other cardiovascular events. The analysis of IBPC vs. SBPC, based on high-quality studies with strong homogeneity, confirms IBPC's superior efficacy in reducing both recurrent stroke and major vascular events.

### Limitations and prospects

Several methodological limitations warrant consideration in this systematic review. Trial design variations across included studies encompass differences in blood pressure measurement methods, patient populations, outcome measure definitions, and follow-up durations. These variations potentially contribute to inter-study heterogeneity, though sensitivity analyses confirm result stability and reliability. The analysis faced constraints regarding blood pressure management protocols. Key variables included methods of blood pressure reduction, antihypertensive drug selection, treatment initiation timing, intervention duration, and stroke recurrence timing. However, limited original data availability prevented detailed subgroup analysis of these factors. Additionally, baseline clinical characteristics showed substantial variation across studies, including age, NIHSS scores, and medical/medication histories. The absence of comprehensive original data precluded subgroup analysis of these important variables.

## Conclusions

Current evidence demonstrates that antihypertensive therapy significantly reduces stroke recurrence and vascular mortality risks compared to control interventions. IBPC provides additional benefits over SBPC in preventing stroke recurrence and major vascular events, with favorable safety profiles and sustained long-term outcomes. While these findings strongly support current treatment strategies, validation through large-scale, well-designed multicenter RCTs remains essential.

## Supplementary Information

Additional file 1.

Additional file 2.

Additional file 3.

## Data Availability

No datasets were generated or analysed during the current study.

## Abbreviations

RCTs: Randomized-controlled trials
RR: Risk ratio
CI: Confidence interval
*P*: Probability
*I*^2^: Index of heterogeneity

## References

[1] Hsu CY, Saver JL, Ovbiagele B, Wu YL, Cheng CY, Lee M. Association between magnitude of differential blood pressure reduction and secondary stroke prevention: a meta-analysis and meta-regression. JAMA Neurol. 2023;80(5):506–15. 10.1001/jamaneurol.2023.0218. 36939729 10.1001/jamaneurol.2023.0218 PMC10028545

[2] Mustanoja S, Putaala J, Gordin D, Tulkki L, Aarnio K, Pirinen J, et al. Acute-phase blood pressure levels correlate with a high risk of recurrent strokes in young-onset ischemic stroke. Stroke. 2016;47(6):1593–8. 10.1161/strokeaha.116.012944. 27217509 10.1161/STROKEAHA.116.012944

[3] GBD 2016 Stroke Collaborators. Global, regional, and national burden of stroke, 1990–2016: a systematic analysis for the Global Burden of Disease Study 2016. Lancet Neurol. 2019;18:439–58. 10.1016/s1474-4422(18)30499-x. 30871944 10.1016/S1474-4422(19)30034-1 PMC6494974

[4] Feigin VL, Norrving B, Mensah GA. Global burden of stroke. Circ Res. 2017;120:439–48. 10.1161/circresaha.116.308413. 28154096 10.1161/CIRCRESAHA.116.308413

[5] Medeiros GC, Roy D, Kontos N, Beach SR. Post-stroke depression: a 2020 updated review. Gen Hosp Psychiatry. 2020;66:70–80. 10.1016/j.genhosppsych.2020.06.011. 32717644 10.1016/j.genhosppsych.2020.06.011

[6] Zonneveld TP, Richard E, Vergouwen MD, Nederkoorn PJ, de Haan R, Roos YB, et al. Blood pressure-lowering treatment for preventing recurrent stroke, major vascular events, and dementia in patients with a history of stroke or transient ischaemic attack. Cochrane Database Syst Rev. 2018;7:Cd007858. 10.1002/14651858.CD007858.pub2. 30024023 10.1002/14651858.CD007858.pub2 PMC6513249

[7] Benavente OR, Coffey CS, Conwit R, Hart RG, McClure LA, Pearce LA, et al. Blood-pressure targets in patients with recent lacunar stroke: the SPS3 randomised trial. Lancet. 2013;38(2):507–15. 10.1016/s0140-6736(13)60852-1. 10.1016/S0140-6736(13)60852-1 PMC3979302 23726159

[8] Mant J, McManus RJ, Roalfe A, Fletcher K, Taylor CJ, Martin U, et al. Different systolic blood pressure targets for people with history of stroke or transient ischaemic attack: PAST-BP (prevention after stroke-blood pressure) randomised controlled trial. BMJ. 2016;352: i708. 10.1136/bmj.i708. 26919870 10.1136/bmj.i708 PMC4770816

[9] Kitagawa K, Yamamoto Y, Arima H, Maeda T, Sunami N, Kanzawa T, et al. Effect of standard vs intensive blood pressure control on the risk of recurrent stroke: a randomized clinical trial and meta-analysis. JAMA Neurol. 2019;76:1309–18. 10.1001/jamaneurol.2019.2167. 31355878 10.1001/jamaneurol.2019.2167 PMC6664380

[10] Chalmers J. Trials on blood pressure-lowering and secondary stroke prevention. Am J Cardiol. 2003;91:3g–8g. 10.1016/s0002-9149(03)00226-1. 12781902 10.1016/s0002-9149(03)00226-1

[11] Sandset EC, Bath PM, Boysen G, Jatuzis D, Kõrv J, Lüders S, et al. The angiotensin-receptor blocker candesartan for treatment of acute stroke (SCAST): a randomised, placebo-controlled, double-blind trial. Lancet. 2011;377:741–50. 10.1016/s0140-6736(11)60104-9. 21316752 10.1016/S0140-6736(11)60104-9

[12] PROGRESS Collaborative Group. Randomised trial of a perindopril-based blood-pressure-lowering regimen among 6,105 individuals with previous stroke or transient ischaemic attack. Lancet. 2001;358:1033–41. 10.1016/s0140-6736(01)06178-5. 11589932 10.1016/S0140-6736(01)06178-5

[13] Trial of secondary prevention with atenolol after transient ischemic attack or nondisabling ischemic stroke. The Dutch TIA Trial Study Group. Stroke. 1993;24:543–48. 10.1161/01.str.24.4.543 10.1161/01.str.24.4.543 8465360

[14] Bath PM, Martin RH, Palesch Y, Cotton D, Yusuf S, Sacco R, et al. Effect of telmisartan on functional outcome, recurrence, and blood pressure in patients with acute mild ischemic stroke: a PRoFESS subgroup analysis. Stroke. 2009;40:3541–6. 10.1161/strokeaha.109.555623. 19797187 10.1161/STROKEAHA.109.555623

[15] Liu L, Wang Z, Gong L, Zhang Y, Thijs L, Staessen JA, et al. Blood pressure reduction for the secondary prevention of stroke: a Chinese trial and a systematic review of the literature. Hypertens Res. 2009;32:1032–40. 10.1038/hr.2009.139. 19798097 10.1038/hr.2009.139

[16] Wright JT Jr, Williamson JD, Whelton PK, Snyder JK, Sink KM, Rocco MV, et al. A randomized trial of intensive versus standard blood-pressure control. N Engl J Med. 2015;373:2103–16. 10.1056/NEJMoa1511939. 26551272 10.1056/NEJMoa1511939 PMC4689591

[17] Yusuf S, Sleight P, Pogue J, Bosch J, Davies R, Dagenais G. Effects of an angiotensin-converting-enzyme inhibitor, ramipril, on cardiovascular events in high-risk patients. N Engl J Med. 2000;342:145–53. 10.1056/nejm200001203420301. 10639539 10.1056/NEJM200001203420301

[18] PATS Collaborating Group. Post-stroke antihypertensive treatment study. A preliminary result. Chin Med J (Engl). 1995;108:710–7. 8575241

[19] Effect of antihypertensive treatment on stroke recurrence. Hypertension-Stroke Cooperative Study Group. Jama. 1974; 229:409–18. 10.1001/jama.1974.03230420021019. 10.1001/jama.1974.03230420021019 4599980

[20] Yusuf S, Diener HC, Sacco RL, Cotton D, Ounpuu S, Lawton WA, et al. Telmisartan to prevent recurrent stroke and cardiovascular events. N Engl J Med. 2008;359:1225–37. 10.1056/NEJMoa0804593. 18753639 10.1056/NEJMoa0804593 PMC2714258

[21] Hasegawa Y, Yamaguchi T, Omae T, Woodward M, Chalmers J. Effects of perindopril-based blood pressure lowering and of patient characteristics on the progression of silent brain infarct: the Perindopril Protection against Recurrent Stroke Study (PROGRESS) CT Substudy in Japan. Hypertens Res. 2004;27:147–56. 10.1291/hypres.27.147. 15080373 10.1291/hypres.27.147

[22] Odden MC, McClure LA, Sawaya BP, White CL, Peralta CA, Field TS, et al. Achieved blood pressure and outcomes in the secondary prevention of small subcortical strokes trial. Hypertension. 2016;67:63–9. 10.1161/hypertensionaha.115.06480. 26553236 10.1161/HYPERTENSIONAHA.115.06480 PMC4679688

[23] Carter AB. Hypotensive therapy in stroke survivors. Lancet. 1970;1:485–9. 10.1016/s0140-6736(70)91577-1. 4190177 10.1016/s0140-6736(70)91577-1

[24] Bath PM, Scutt P, Blackburn DJ, Ankolekar S, Krishnan K, Ballard C, et al. Intensive versus guideline blood pressure and lipid lowering in patients with previous stroke: main results from the pilot “prevention of decline in cognition after stroke trial” (PODCAST) randomised controlled trial. PLoS ONE. 2017;12: e0164608. 10.1371/journal.pone.0164608. 28095412 10.1371/journal.pone.0164608 PMC5240987

[25] Eriksson S, Olofsson B-O, Wester P-O. Atenolol in secondary prevention after stroke. Cerebrovasc Dis. 1995;5(1):21–5. 10.1159/000107813.

[26] Kitagawa K, Arima H, Yamamoto Y, Ueda S, Rakugi H, Kohro T, et al. Intensive or standard blood pressure control in patients with a history of ischemic stroke: RESPECT post hoc analysis. Hypertens Res. 2022;45:591–601. 10.1038/s41440-022-00862-y. 35241817 10.1038/s41440-022-00862-y

[27] Gueyffier F, Boissel JP, Boutitie F, Pocock S, Coope J, Cutler J, et al. Effect of antihypertensive treatment in patients having already suffered from stroke. Gathering the evidence. The INDANA (INdividual Data ANalysis of Antihypertensive intervention trials) Project Collaborators. Stroke. 1997;28:2557–62. 10.1161/01.str.28.12.2557. 9412649 10.1161/01.str.28.12.2557

[28] Schrader J, Lüders S, Kulschewski A, Berger J, Zidek W, Treib J, et al. The ACCESS study: evaluation of acute candesartan cilexetil therapy in stroke survivors. Stroke. 2003;34(7):1699–703. 10.1161/01.Str.0000075777.18006.89. 12817109 10.1161/01.STR.0000075777.18006.89

[29] Tanahashi N, Nakagawara J, Okada Y, Minematsu K. Candesartan cilexetil in the management of blood pressure for acute and recurrent stroke in Japan: the Challenge-Stroke study. Expert Rev Cardiovasc Ther. 2011;9(9):1115–26. 10.1586/erc.11.132. 21932954 10.1586/erc.11.132

