# Supplementary material for: Antihypertensive strategies for the prevention of secondary stroke: a systematic review and meta-analysis
Source: Eur J Med Res. 2025 Jan 9;30:18. doi: 10.1186/s40001-024-02226-3 (PMC11715515; doi:10.1186/s40001-024-02226-3)
Supplement: Supplementary file 1 — Additional file 1. [file 40001_2024_2226_MOESM1_ESM.docx]

**Table S1.** Literature search strategy.

**1.Search strategy in Pubmed**

|  | Mesh | Title/Abstract |
| --- | --- | --- |
| #1 | Stroke (168052) | Stroke OR strokes OR Cerebrovascular Accident OR Cerebrovascular Accidents OR CVA OR CVAs OR Cerebrovascular Apoplexy OR Brain Vascular Accident OR Cerebral Stroke OR Cerebral Strokes OR Acute Stroke OR Acute Strokes OR Acute Cerebrovascular Accident OR Acute Cerebrovascular Accidents (317010) |
| #2 | Ischemic Attack, Transient (21720) | Ischemic Attack, Transient OR TIA OR TIAs OR Transient Ischemic Attack OR Transient Ischemic Attacks OR Brain TIA OR Transient Brainstem Ischemia OR Transient Cerebral Ischemia OR Transient Cerebral Ischemias (21096) |
| #3 | #1 OR #2(369730) | |
| #4 | Antihypertensive Agents OR Angiotensin II Type 1 Receptor Blockers OR Guanfacine OR Betaxolol OR Bepridil OR Minoxidil OR Rauwolfia OR Hexamethonium Compounds OR Amlodipine Besylate, Olmesartan Medoxomil Drug Combination OR 1-hexadecyl-2-acetyl-glycero-3-phosphocholine OR medullipin I OR 3,3,5,5-tetramethyl-4-piperidone OR Calcium Channel Blockers OR Clonidine OR Debrisoquin OR Germine Acetates OR Enalapril (138415) |  |
| #5 | Hypertension/prevention and control OR Hypertension/therapy (118247) |  |
| #6 | #4 OR #5 (208492) | |
| #7 |  | antihypertensive OR Hypertension therapy OR (Hypertension control OR Antihypertensive Agent OR Anti-Hypertensive Agent OR Anti Hypertensive Drug OR Antihypertensive Drug OR Antihypertensive Drugs OR Antihypertensives OR Anti-Hypertensive Agents OR Anti Hypertensive Agents OR Anti-Hypertensive Drugs OR Anti Hypertensive Drugs OR Anti-Hypertensives OR Anti Hypertensives OR Anti-Hypertensive OR Anti Hypertensive OR Antihypertensive OR Hypertension control OR Blood Pressure control OR Intensive Blood Pressure Control OR antihypertensive therapy OR vasodilator OR adrenergic agonist OR diuretic OR angiotensin receptor antagonist OR dipeptidyl carboxypeptidase inhibitor OR calcium channel blockers OR alpha adrenergic receptor blocking agent OR hydralazine OR losartan (139047) |
| #8 | #6 OR #7 (287798) | |
| #9 | #3 AND #8 (13847) | |
| #10 | Recurrence (199303) | Recurrence OR Recurrences OR Recrudescence OR Recrudescences OR Relapse OR Relapses (526005) |
| #11 | #9 AND #10 (456) | |

**2.Search strategy in Cochrane**

|  | MeSH descriptor | ti,ab,kw |
| --- | --- | --- |
| #1 | Stroke (14102) |  |
| #2 | Ischemic Attack, Transient (950) |  |
| #3 |  | Stroke OR strokes OR Cerebrovascular Accident OR Cerebrovascular Accidents OR CVA(69711) |
| #4 |  | CVAs OR Cerebrovascular Apoplexy OR Brain Vascular Accident OR Cerebral Stroke OR Cerebral Strokes (7807) |
| #5 |  | Acute Stroke OR Acute Strokes OR Acute Cerebrovascular Accident OR Acute Cerebrovascular Accidents OR Ischemic Attack, Transient (21007) |
| #6 |  | TIA OR TIAs OR Transient Ischemic Attack OR Transient Ischemic Attacks OR Brain TIA(4648) |
| #7 |  | Transient Brainstem Ischemia OR Transient Cerebral Ischemia OR Transient Cerebral Ischemias (406) |
| #8 | #1 or #2 or #3 or #4 or #5 or #6 or #7(70948) | |
| #9 | Antihypertensive Agents (9547) |  |
| #10 |  | antihypertensive OR Hypertension therapy OR Hypertension control OR Antihypertensive Agent OR Anti-Hypertensive Agent(57132) |
| #11 |  | Anti Hypertensive Drug OR Antihypertensive Drug OR Antihypertensive Drugs OR Antihypertensives OR Anti-Hypertensive Agents (18263) |
| #12 |  | Anti Hypertensive Agents OR Anti-Hypertensive Drugs OR Anti Hypertensive Drugs OR Anti-Hypertensives OR Anti Hypertensives(1067) |
| #13 |  | Anti-Hypertensive OR Anti Hypertensive OR Antihypertensive OR Hypertension control OR Blood Pressure control(70273) |
| #14 |  | Intensive Blood Pressure Control OR antihypertensive therapy OR vasodilator OR adrenergic agonist OR diuretic(32201) |
| #15 |  | angiotensin receptor antagonist OR dipeptidyl carboxypeptidase inhibitor OR calcium channel blockers OR alpha adrenergic receptor blocking agent OR hydralazine(8712) |
| #16 |  | losartan (2698) |
| #17 | #9 or #10 or #11 or #12 or #13 or #14 or #15 or #16 (103461) | |
| #18 | Recurrence(14363) |  |
| #19 |  | Recurrence OR Recurrences OR Recrudescence OR Recrudescences OR Relapse（81090) |
| #20 |  | Relapses(5777) |
| #21 | #18 or #19 or #20 (82689) | |
| #22 | #8 and #17 and #21 (410) | |

**3.Search strategy in Embase**

|  | exp | ab,ti |
| --- | --- | --- |
| #1 | cerebrovascular accident **(**409900) |  |
| #2 | transient ischemic attack (46817) |  |
| #3 | #1 OR #2 (431432) | |
| #4 |  | stroke OR strokes OR cerebrovascular accident OR cerebrovascular accidents OR cva OR cvas OR cerebrovascular apoplexy OR brain vascular accident OR cerebral stroke OR cerebral strokes OR acute strokeOR acute strokes OR acute cerebrovascular accident OR acute cerebrovascular accidents OR ischemic attack, transient OR tia OR tiasi OR transient ischemic attack OR transient ischemic attacks OR brain tia OR transient brainstem ischemia OR transient cerebral ischemia OR transient cerebral ischemias (504231) |
| #5 | #3 OR #4 (619009) | |
| #6 | antihypertensive (940213) |  |
| #7 | resistant hypertension(57045704) |  |
| #8 |  | hypertension therapy OR antihypertensive agent OR anti-hypertensive agent OR anti hypertensive drug OR antihypertensive drug OR antihypertensive drugs OR antihypertensives OR anti-hypertensive agents OR anti hypertensive agents OR anti-hypertensive drugs OR anti hypertensive drugs OR anti hypertensives OR anti hypertensive OR antihypertensive OR hypertension control OR blood pressure control OR intensive blood pressure control OR antihypertensive therapy OR vasodilator OR adrenergic agonist OR diuretic OR angiotensin receptor antagonist OR dipeptidyl carboxypeptidase inhibitor OR calcium channel blockers OR alpha adrenergic receptor blocking agent OR hydralazine OR losartan (193963) |
| #9 | #6 OR #7 OR #8(1013006) | |
| #10 | recurrent disease (210463) |  |
| #11 |  | recurrence OR recurrences OR recrudescence OR recrudescences OR relapse:ab,ti OR relapses (819764) |
| #12 | #10 OR #11(940971) | |
| #13 | #3 OR #5 (619009) | |
| #14 | #9 AND #12 AND #13 (2045) | |

1. **Search strategy in Web of Science**

|  | Topic |
| --- | --- |
| #1 | Stroke OR strokes OR Cerebrovascular Accident OR Cerebrovascular Accidents OR CVA OR CVAs OR Cerebrovascular Apoplexy OR Brain Vascular Accident OR Cerebral Stroke OR Cerebral Strokes OR Acute Stroke OR Acute Strokes OR Acute Cerebrovascular Accident OR Acute Cerebrovascular Accidents (846968) |
| #2 | Ischemic Attack, Transient OR TIA OR TIAs OR Transient Ischemic Attack OR Transient Ischemic Attacks OR Brain TIA OR Transient Brainstem Ischemia OR Transient Cerebral Ischemia OR Transient Cerebral Ischemias (64758) |
| #3 | antihypertensive OR Hypertension therapy OR Hypertension control OR Antihypertensive Agent OR Anti-Hypertensive Agent OR Anti Hypertensive Drug OR Antihypertensive Drug OR Antihypertensive Drugs OR Antihypertensives OR Anti-Hypertensive Agents OR Anti Hypertensive Agents OR Anti-Hypertensive Drugs OR Anti Hypertensive Drugs OR Anti-Hypertensives OR Anti Hypertensives OR Anti-Hypertensive OR Anti Hypertensive OR Antihypertensive OR Hypertension control OR Blood Pressure control OR Intensive Blood Pressure Control OR antihypertensive therapy OR vasodilator OR adrenergic agonist OR diuretic OR angiotensin receptor antagonist OR dipeptidyl carboxypeptidase inhibitor OR calcium channel blockers OR alpha adrenergic receptor blocking agent OR hydralazine OR losartan (1073506) |
| #4 | Recurrence OR Recurrences OR Recrudescence OR Recrudescences OR Relapse OR Relapses (1018318) |
| #5 | #2 OR #1 (876287) |
| #6 | #3AND #4 AND #5(1990) |
