# Supplementary material for: Antihypertensive strategies for the prevention of secondary stroke: a systematic review and meta-analysis
Source: Eur J Med Res. 2025 Jan 9;30:18. doi: 10.1186/s40001-024-02226-3 (PMC11715515; doi:10.1186/s40001-024-02226-3)
Supplement: Supplementary file 2 — Additional file 2. [file 40001_2024_2226_MOESM2_ESM.doc]

**Table S2.** Meta-analysis regression.

|  | **Coef.** | **Std.Err.** | **Z** | **P>│z│** | **[95% Conf. Interval]** |
| --- | --- | --- | --- | --- | --- |
| -cons | .9692718 | .017932 | 54.05 | 0.000 | .9341259 1.004418 |
