# Supplementary material for: Antihypertensive strategies for the prevention of secondary stroke: a systematic review and meta-analysis
Source: Eur J Med Res. 2025 Jan 9;30:18. doi: 10.1186/s40001-024-02226-3 (PMC11715515; doi:10.1186/s40001-024-02226-3)
Supplement: Supplementary file 3 — Additional file 3. [file 40001_2024_2226_MOESM3_ESM.doc]

Table S3. All-cause Mortality Egger's test

| **Std_Eff** | **Coef.** | **Std. Err.** | **t** | **P>t** | **[95% Conf.** | **Interval]** |
| --- | --- | --- | --- | --- | --- | --- |
| slope | -.1330173 | .0787023 | -1.690.129 | -.3145051 | .0484704 |  |
| bias | .495754 | .8808549 | 0.56 | 0.589 | -1.535501 | 2.527009 |
